# Supplementary material for: A Likelihood Approach for Real-Time Calibration of Stochastic Compartmental Epidemic Models
Source: PLoS Comput Biol. 2017 Jan 17;13(1):e1005257. doi: 10.1371/journal.pcbi.1005257 (PMC5240920; doi:10.1371/journal.pcbi.1005257)
Supplement: S1 File — (TAR.GZ) [file pcbi.1005257.s014.tar.gz › HSPH_Online-SI-Revision/EnKF07/n100-mild/n100-mild_EnKF_table-8.pdf]

1	R0	{1.24125, 3.38982}	{2.51433, 4.57197}	{{2.00396, 1.25886}, {3., 7.53409}}	{103.661, 69.926}	inf	218.	2276.58	{736.649, 2260.7}	947.243	0.807556	newly	544.	2868.56	{680., 4294.}	431.629	0.488927	Reff	1.2258	0.540851	{0.142181, 1.32477}	67.7407	ar	34 926.	181 016.	{165 791., 217 076.}	419.308
2	R0	{1.29554, 4.51878}	{2.51219, 6.96406}	{{1.45794, 3.36749}, {3., 10.2529}}	{93.9104, 67.8585}	inf	496.	3203.48	{1486.74, 2938.45}	548.369	0.5583	newly	1039.	3379.15	{742., 3349.}	247.86	0.289571	Reff	1.26727	0.52842	{0.0258367, 0.967186}	64.4287	ar	42 828.	181 213.	{170 099., 197 719.}	323.117
3	R0	{1.2443, 3.64292}	{2.21789, 4.2158}	{{1.05399, 2.64687}, {3., 4.9889}}	{85.4542, 28.6823}	inf	288.	13 342.8	{5040.26, 22 295.3}	4532.9	1.53183	newly	581.	14 270.3	{5218., 28 971.}	2356.16	1.23288	Reff	1.22536	0.338871	{0.0438069, 0.613599}	72.3452	ar	37 087.	161 468.	{94 801., 196 624.}	335.783
4	R0	{1.38465, 3.43331}	{2.05786, 9.96719}	{{0.769878, 3.8806}, {3., 17.0495}}	{65.2355, 197.86}	inf	73.	25 004.6	{8679.36, 46 134.}	34 152.8	2.40271	newly	229.	22 157.1	{5131., 46 226.}	9575.59	1.80832	Reff	1.38185	1.22393	{0.175013, 2.22219}	43.6129	ar	49 511.	127 749.	{81 074., 186 455.}	158.513
5	R0	{1.25409, 4.1068}	{2.07658, 4.23386}	{{1.05998, 2.64648}, {3., 4.99505}}	{74.4403, 26.6892}	inf	293.	12 603.8	{5597.77, 22 439.7}	4201.64	1.49675	newly	618.	14 459.4	{6286., 34 792.}	2239.71	1.19672	Reff	1.23932	0.381746	{0.0434933, 1.01942}	70.4201	ar	38 571.	159 158.	{78 422., 194 499.}	312.637
6	R0	{1.28821, 4.2016}	{2.54411, 4.75808}	{{2.00344, 1.25719}, {3., 7.50456}}	{97.9801, 51.0966}	inf	415.	2274.83	{751.543, 2821.95}	453.862	0.541623	newly	847.	3360.9	{681., 4527.}	311.981	0.373979	Reff	1.26157	0.581088	{0.178859, 1.68507}	66.9776	ar	41 468.	181 558.	{165 109., 246 171.}	337.826
7	R0	{1.24369, 2.56007}	{2.12504, 6.02342}	{{0.847841, 2.17253}, {3., 10.0939}}	{94.3516, 142.247}	inf	56.	31 760.8	{12 429.5, 61 701.4}	56 615.7	2.66401	newly	202.	39 021.2	{11 423., 86 653.}	19 217.4	2.15578	Reff	1.24058	1.07239	{0.401943, 1.57451}	31.5596	ar	38 603.	137 946.	{84 496., 204 702.}	257.967
8	R0	{1.232, 3.53942}	{2.43287, 6.52938}	{{1.46151, 3.37462}, {3., 10.2749}}	{97.4731, 88.518}	inf	337.	3653.09	{1505.84, 8808.19}	986.85	0.790166	newly	787.	4739.8	{768., 12 744.}	505.787	0.402555	Reff	1.2117	0.561027	{0.024819, 1.46125}	62.7987	ar	34 555.	186 043.	{170 824., 245 984.}	438.398
9	R0	{1.36262, 3.85631}	{2.48341, 7.79086}	{{2.15979, 4.3167}, {3., 14.7091}}	{84.5759, 102.806}	inf	149.	23 306.2	{4040.07, 55 675.3}	15 541.7	2.02973	newly	418.	23 666.3	{6144., 69 610.}	5561.8	1.56421	Reff	1.35645	1.04951	{0.454068, 2.18571}	48.9971	ar	48 410.	158 864.	{107 325., 224 680.}	228.164
10	R0	{1.28702, 4.67968}	{2.20047, 8.99138}	{{0.406415, 2.69672}, {3., 13.6626}}	{102.401, 115.834}	inf	588.	4294.4	{2752.17, 2752.17}	630.341	0.71246	newly	1085.	2996.05	{1155., 4029.}	176.161	0.281794	Reff	1.24853	0.381551	{0.0766196, 0.634973}	70.2678	ar	41 092.	176 609.	{170 418., 181 204.}	329.788
11	R0	{1.35752, 3.47129}	{2.08217, 4.04893}	{{1.05031, 2.64597}, {3., 5.00527}}	{69.2178, 27.1018}	inf	93.	13 798.1	{3959.9, 40 241.1}	14 736.7	1.98645	newly	286.	15 437.2	{4153., 51 967.}	5297.63	1.51736	Reff	1.35065	0.356519	{0.0593477, 0.620393}	73.6039	ar	44 669.	161 633.	{131 723., 205 862.}	261.846
12	R0	{1.35929, 5.83647}	{2.36266, 9.16313}	{{0.4065, 2.69846}, {3., 13.6941}}	{99.0555, 84.9333}	inf	673.	4326.55	{2777.31, 2777.31}	542.874	0.662299	newly	1048.	2972.92	{1148., 3969.}	183.676	0.29543	Reff	1.3216	0.388438	{0.0743689, 0.650888}	71.2867	ar	46 715.	177 337.	{170 770., 181 930.}	279.614
13	R0	{1.28019, 3.29908}	{2.54232, 4.90792}	{{1.81446, 1.24485}, {3., 12.8151}}	{98.5891, 103.475}	inf	93.	23 610.2	{4066.18, 56 637.}	25 287.3	2.21046	newly	305.	28 727.2	{2421., 73 395.}	9319.89	1.74159	Reff	1.27548	0.634787	{0.255567, 1.2776}	52.7951	ar	40 941.	150 033.	{84 890., 247 533.}	266.462
14	R0	{1.36661, 3.48318}	{2.1691, 6.00713}	{{0.847664, 2.1725}, {3., 10.0928}}	{84.1451, 91.2755}	inf	101.	31 950.2	{12 424.2, 61 681.8}	31 533.9	2.40956	newly	296.	40 191.3	{11 375., 86 754.}	13 478.1	1.99546	Reff	1.36217	1.10203	{0.40208, 1.63486}	32.4223	ar	48 672.	139 341.	{84 479., 204 478.}	187.19
15	R0	{1.35865, 3.84123}	{1.96614, 4.39971}	{{1.04916, 2.64582}, {3., 5.00628}}	{62.9695, 31.3207}	inf	112.	15 429.9	{5007.24, 49 115.2}	13 676.7	1.94165	newly	273.	16 477.8	{4200., 56 175.}	5935.83	1.55569	Reff	1.35217	0.356233	{0.0457471, 0.609579}	74.7489	ar	48 598.	162 674.	{111 369., 219 621.}	234.852
16	R0	{1.22879, 3.98329}	{2.35475, 8.98765}	{{0.410232, 2.70653}, {3., 13.6906}}	{116.939, 142.905}	inf	457.	4595.49	{2780.43, 2780.43}	905.578	0.841118	newly	913.	3200.69	{1149., 4017.}	250.568	0.372746	Reff	1.20087	0.385633	{0.0758905, 0.646535}	68.8614	ar	34 405.	177 581.	{171 036., 181 944.}	416.148
17	R0	{1.34925, 3.64524}	{2.44889, 7.94267}	{{2.15899, 4.31391}, {3., 14.7098}}	{85.3079, 118.598}	inf	135.	23 382.2	{6631.45, 56 593.6}	17 220.2	2.07806	newly	291.	22 572.3	{6177., 59 515.}	7656.79	1.70679	Reff	1.34265	0.997901	{0.425434, 2.16937}	48.1575	ar	46 871.	157 330.	{107 482., 224 410.}	236.236
18	R0	{1.33054, 2.25275}	{1.75976, 8.5275}	{{0.388719, 2.00079}, {3., 18.108}}	{71.3508, 288.502}	inf	11.	6429.76	{1231.97, 11 976.3}	58 352.4	2.61991	newly	52.	20 393.9	{1608., 67 493.}	39 119.	2.183	Reff	1.33009	1.61631	{0.320456, 2.73832}	65.0216	ar	44 336.	69 331.3	{15 793., 106 329.}	89.8554
19	R0	{1.28394, 3.69859}	{2.42403, 6.5924}	{{1.46304, 3.3749}, {3., 10.2607}}	{88.796, 85.4819}	inf	236.	4320.73	{1489.44, 8811.19}	1736.38	0.945708	newly	569.	5115.57	{742., 25 401.}	805.26	0.554495	Reff	1.26314	0.583463	{0.0353349, 1.46238}	61.7662	ar	39 558.	186 783.	{169 738., 244 911.}	372.176
20	R0	{1.2883, 2.26493}	{1.73238, 7.85575}	{{0.31596, 2.00079}, {3., 16.7628}}	{75.7444, 259.956}	inf	9.	6831.7	{1102.62, 13 313.1}	75 807.8	2.71825	newly	36.	22 167.2	{1987., 72 659.}	61 475.4	2.40631	Reff	1.28811	1.57201	{0.269684, 2.92656}	70.0227	ar	40 768.	67 965.1	{15 849., 106 373.}	99.3029
21	R0	{1.34098, 2.78621}	{1.68123, 8.29275}	{{0.372896, 2.00079}, {3., 17.6864}}	{71.2462, 221.684}	inf	10.	7208.23	{1103.89, 14 487.9}	71 982.3	2.66635	newly	66.	19 728.8	{786., 58 897.}	29 792.1	2.04995	Reff	1.34068	1.48941	{0.248433, 2.73861}	64.3105	ar	44 605.	65 532.8	{15 376., 105 502.}	87.0443
22	R0	{1.33783, 5.95911}	{2.38717, 9.16426}	{{0.404715, 2.69796}, {3., 13.6969}}	{102.164, 82.2315}	inf	837.	4354.16	{2779.96, 2779.96}	420.211	0.568301	newly	1123.	3002.09	{1155., 3991.}	167.427	0.269242	Reff	1.29617	0.396344	{0.0737972, 0.651311}	70.1363	ar	45 418.	177 445.	{170 788., 181 991.}	290.694
23	R0	{1.33359, 3.3069}	{2.55341, 4.87049}	{{1.81451, 1.24177}, {3., 12.8165}}	{92.0689, 103.347}	inf	97.	24 613.5	{4845.87, 59 170.6}	25 275.1	2.21309	newly	260.	30 122.2	{2392., 73 525.}	11 486.7	1.83475	Reff	1.32804	0.646623	{0.255153, 1.2775}	53.1162	ar	46 245.	152 219.	{84 956., 247 933.}	229.934
24	R0	{1.30588, 2.56123}	{2.15116, 9.23888}	{{0.541273, 2.1087}, {3., 12.9319}}	{91.0888, 268.275}	inf	22.	18 025.1	{2682.98, 40 602.5}	81 832.5	2.73424	newly	124.	23 365.1	{3923., 81 662.}	18 745.3	2.00067	Reff	1.30531	1.54865	{0.237684, 2.99997}	58.5329	ar	42 383.	116 340.	{50 347., 188 002.}	176.803
25	R0	{1.29813, 4.51849}	{2.25993, 9.15944}	{{0.412223, 2.70586}, {3., 13.7166}}	{102.74, 125.153}	inf	361.	4899.02	{2776.28, 2776.28}	1257.07	0.941915	newly	633.	3260.78	{1143., 4011.}	415.131	0.527157	Reff	1.27199	0.368358	{0.0754364, 0.564053}	71.0407	ar	43 371.	177 352.	{170 969., 174 740.}	308.918
26	R0	{1.33231, 3.99953}	{2.53632, 4.63678}	{{2.00438, 1.26762}, {3., 7.52139}}	{91.836, 54.0503}	inf	222.	2171.2	{753.222, 2317.05}	881.403	0.800299	newly	479.	2771.66	{678., 4263.}	482.668	0.541194	Reff	1.3147	0.555682	{0.147408, 1.5559}	68.1594	ar	44 733.	182 501.	{165 120., 219 053.}	307.978
27	R0	{1.30555, 4.61445}	{2.23564, 9.27019}	{{0.415809, 2.71376}, {3., 13.7482}}	{101.217, 124.763}	inf	301.	5185.01	{2833.79, 2833.79}	1622.59	1.03582	newly	599.	3438.29	{1162., 4111.}	474.005	0.561139	Reff	1.2885	0.373371	{0.0768353, 0.569101}	71.0228	ar	42 624.	178 766.	{171 124., 183 693.}	319.402
28	R0	{1.26819, 5.34969}	{2.5033, 7.02612}	{{1.4599, 3.37242}, {3., 10.2736}}	{97.3918, 56.697}	inf	559.	3269.88	{1484.73, 3912.78}	490.284	0.523279	newly	884.	3399.43	{728., 3338.}	299.598	0.313867	Reff	1.2407	0.494869	{0.0249386, 0.871783}	66.771	ar	38 946.	181 155.	{170 023., 192 778.}	365.144
29	R0	{1.19925, 3.00394}	{2.04436, 4.23498}	{{0.0849546, 0.940417}, {3., 5.81981}}	{115.057, 75.2826}	inf	185.	5335.15	{548.088, 16 936.}	2783.87	1.1661	newly	516.	6423.95	{483., 16 773.}	1150.72	0.745572	Reff	1.18521	0.303234	{0.0123138, 0.452943}	75.3769	ar	31 727.	170 655.	{157 402., 220 309.}	437.885
30	R0	{1.35749, 3.75483}	{2.40095, 4.98918}	{{1.76853, 1.23598}, {3., 12.8232}}	{76.8669, 88.59}	inf	91.	27 320.7	{4871.72, 58 818.8}	29 922.7	2.29576	newly	259.	30 833.6	{4949., 73 697.}	11 804.9	1.86363	Reff	1.35287	0.631046	{0.25555, 1.36582}	55.2435	ar	48 244.	159 287.	{69 875., 257 507.}	230.221
31	R0	{1.27131, 4.2161}	{2.46589, 4.63874}	{{1.19903, 1.27815}, {3., 7.53807}}	{95.6186, 56.1217}	inf	214.	1714.16	{471.245, 2249.19}	704.285	0.742231	newly	451.	2691.91	{587., 4233.}	505.39	0.504973	Reff	1.25808	0.492786	{0.18031, 1.05181}	68.6343	ar	38 299.	176 528.	{165 643., 195 645.}	362.078
32	R0	{1.31827, 6.18119}	{2.25736, 9.03757}	{{0.40363, 2.69624}, {3., 13.6657}}	{103.107, 76.6411}	inf	869.	4576.26	{2772.52, 2772.52}	426.612	0.560623	newly	1107.	3185.61	{1151., 4025.}	187.878	0.285823	Reff	1.27462	0.383023	{0.0746578, 0.649422}	70.7209	ar	45 262.	178 157.	{171 029., 182 216.}	293.614
33	R0	{1.20398, 1.35167}	{1.71179, 8.25938}	{{0.372931, 2.00079}, {3., 18.0884}}	{81.0159, 513.886}	inf	3.	6730.28	{1104.73, 11 974.8}	224 243.	3.1419	newly	20.	19 706.	{765., 51 874.}	98 429.9	2.57072	Reff	1.20375	1.56441	{0.297289, 2.7385}	73.1422	ar	33 329.	67 144.5	{15 261., 104 893.}	130.256
34	R0	{1.32776, 3.146}	{2.4853, 7.62459}	{{2.16056, 4.32489}, {3., 14.7099}}	{89.6565, 143.361}	inf	86.	23 560.	{6627.28, 41 209.2}	27 295.4	2.30744	newly	292.	26 206.8	{5762., 71 712.}	8874.95	1.74627	Reff	1.32356	0.967479	{0.348903, 2.1358}	51.3971	ar	44 872.	153 039.	{83 411., 234 863.}	241.056
35	R0	{1.279, 1.11336}	{1.69442, 8.35489}	{{0.386741, 1.62979}, {3., 16.7565}}	{68.036, 653.572}	inf	8.	8244.03	{1564.65, 17 135.4}	102 950.	2.85395	newly	51.	22 530.1	{2042., 70 400.}	44 076.6	2.2578	Reff	1.27844	1.48594	{0.328351, 2.71776}	61.9369	ar	41 033.	68 308.2	{15 946., 106 433.}	95.086
36	R0	{1.20029, 1.94717}	{1.73698, 8.04864}	{{0.37286, 2.00079}, {3., 17.6872}}	{81.759, 316.961}	inf	9.	7086.32	{1103.04, 13 935.3}	78 636.9	2.69893	newly	29.	20 718.4	{1069., 54 309.}	71 342.8	2.4473	Reff	1.20009	1.54414	{0.248397, 2.9115}	74.2886	ar	33 234.	67 853.7	{15 805., 105 434.}	130.539
37	R0	{1.33216, 3.69685}	{2.07422, 4.28332}	{{0.0866482, 0.938541}, {3., 5.82273}}	{98.7101, 59.9443}	inf	191.	4861.54	{920.079, 13 913.6}	2445.31	1.18697	newly	501.	5562.17	{717., 13 753.}	1013.43	0.772377	Reff	1.3191	0.276717	{0.0126508, 0.452814}	79.0224	ar	45 235.	170 970.	{159 120., 190 041.}	277.96
38	R0	{1.25434, 2.04314}	{1.75144, 8.54045}	{{0.388737, 2.00079}, {3., 18.1076}}	{76.2258, 321.403}	inf	23.	7512.94	{1156.26, 13 317.1}	32 565.	2.33288	newly	145.	21 074.2	{1856., 67 949.}	14 434.7	1.76553	Reff	1.25374	1.58292	{0.320461, 2.73826}	68.1241	ar	38 662.	69 793.2	{15 797., 106 455.}	109.918
39	R0	{1.27466, 3.0741}	{2.06879, 6.16357}	{{0.849868, 2.17228}, {3., 10.0906}}	{83.5559, 115.755}	inf	125.	30 728.2	{15 112.7, 52 615.}	24 482.5	2.3001	newly	445.	36 125.4	{13 706., 69 833.}	8018.06	1.80584	Reff	1.2688	1.04392	{0.404038, 1.5547}	32.3982	ar	40 181.	140 058.	{84 551., 199 471.}	248.568
40	R0	{1.33045, 2.68154}	{2.37714, 8.66162}	{{0.783613, 2.10861}, {3., 13.6273}}	{92.5784, 233.56}	inf	25.	18 292.4	{6674.27, 40 612.3}	73 069.7	2.75151	newly	85.	31 096.	{4772., 84 066.}	36 483.5	2.33924	Reff	1.32907	1.81684	{0.298122, 2.99997}	62.8757	ar	45 951.	129 901.	{67 602., 182 804.}	184.113
41	R0	{1.37003, 2.83984}	{1.67061, 8.08624}	{{0.274064, 2.00079}, {3., 17.6866}}	{68.6195, 212.097}	inf	9.	6491.83	{1103.68, 11 973.8}	72 031.4	2.67134	newly	52.	19 834.5	{808., 54 962.}	38 043.3	2.15751	Reff	1.36976	1.51889	{0.248424, 2.75893}	63.8054	ar	48 926.	65 515.4	{15 630., 104 812.}	75.6306
42	R0	{1.3003, 2.94885}	{2.19165, 5.49432}	{{0.848379, 2.17265}, {3., 10.0949}}	{89.4745, 100.535}	inf	51.	31 528.1	{7725.72, 53 973.9}	61 719.8	2.63449	newly	214.	40 153.6	{12 211., 86 486.}	18 665.6	2.10436	Reff	1.29797	1.07478	{0.403751, 1.55533}	32.3777	ar	44 237.	137 561.	{84 691., 197 397.}	211.511
43	R0	{1.33756, 2.92653}	{1.67879, 8.25895}	{{0.388683, 2.00079}, {3., 18.0878}}	{69.9113, 207.934}	inf	19.	6898.81	{1155., 13 226.2}	36 209.5	2.37488	newly	61.	18 414.5	{1138., 51 564.}	30 087.7	2.08184	Reff	1.33705	1.50505	{0.297288, 2.71838}	63.4551	ar	47 342.	65 051.7	{15 839., 104 743.}	76.7728
44	R0	{1.35801, 2.92276}	{2.15091, 9.09874}	{{0.579, 2.10868}, {3., 12.879}}	{82.6241, 223.434}	inf	11.	17 552.3	{2563.17, 40 602.3}	159 466.	3.01116	newly	66.	23 796.4	{2893., 81 723.}	35 956.1	2.25316	Reff	1.35749	1.56548	{0.274626, 2.99997}	53.248	ar	47 940.	116 724.	{50 380., 188 009.}	148.057
45	R0	{1.31342, 4.02649}	{2.5285, 4.70517}	{{1.77236, 1.23614}, {3., 12.7637}}	{92.5127, 78.659}	inf	222.	24 322.9	{4841.9, 57 789.5}	10 857.5	1.84996	newly	477.	27 918.8	{2371., 73 022.}	5754.52	1.54758	Reff	1.30142	0.575207	{0.131712, 1.0279}	57.7216	ar	42 995.	156 061.	{92 781., 247 804.}	262.975
46	R0	{1.37919, 3.75889}	{2.50037, 6.93614}	{{2.15951, 4.30211}, {3., 10.6448}}	{83.5161, 86.7632}	inf	78.	23 897.1	{8183.35, 55 668.7}	30 537.4	2.36118	newly	193.	25 290.3	{6040., 59 733.}	13 003.8	1.96027	Reff	1.37534	0.913037	{0.368652, 2.11705}	51.0449	ar	49 157.	148 856.	{83 056., 218 520.}	203.665
47	R0	{1.37458, 3.56117}	{2.55702, 7.29751}	{{2.16002, 4.31564}, {3., 14.702}}	{88.7103, 106.271}	inf	90.	21 758.9	{6619.46, 41 102.5}	24 076.6	2.25201	newly	236.	22 275.8	{5731., 58 053.}	9338.89	1.80337	Reff	1.37118	0.89349	{0.367648, 1.82951}	49.3815	ar	48 478.	144 835.	{83 234., 215 593.}	198.765
48	R0	{1.33393, 3.78296}	{2.47173, 6.45583}	{{1.46511, 3.38824}, {3., 10.2974}}	{85.2967, 77.2173}	inf	172.	2951.12	{1492.86, 8756.99}	1617.16	1.0427	newly	441.	4564.98	{766., 25 008.}	936.812	0.625656	Reff	1.3221	0.590862	{0.206131, 1.46397}	60.7319	ar	44 885.	183 646.	{170 094., 244 871.}	309.148
49	R0	{1.24918, 2.39046}	{2.13972, 5.92868}	{{0.847823, 2.17244}, {3., 10.0891}}	{94.2442, 152.575}	inf	41.	32 494.3	{12 353.4, 61 585.2}	79 154.3	2.80835	newly	241.	40 935.5	{11 200., 86 708.}	16 885.7	2.09371	Reff	1.2448	1.0888	{0.402656, 1.63591}	31.9586	ar	35 969.	138 699.	{84 575., 204 586.}	286.115
50	R0	{1.28281, 2.3032}	{2.14723, 9.70563}	{{0.639294, 7.65573}, {3., 17.0459}}	{83.3158, 325.079}	inf	28.	20 522.6	{4243.24, 34 170.3}	73 195.1	2.73332	newly	129.	19 475.6	{4235., 28 156.}	14 997.4	1.97954	Reff	1.28046	1.22442	{0.339625, 1.86256}	39.9213	ar	40 223.	125 668.	{81 953., 172 181.}	212.671
